# Self-compassion in medical students: a pilot study of its association with professionalism pressure

**DOI:** 10.1186/s12909-021-02930-2

**Published:** 2021-09-22

**Authors:** Miroslav Světlák, Šárka Daňhelová, Barbora Kóša, Alena Slezáčková, Rastislav Šumec

**Affiliations:** 1 Department of Psychology and Psychosomatics, Faculty of Medicine, Masaryk University, Brno, Czechia; 2 Department of Psychiatry, Faculty of Medicine, Masaryk University, Brno, Czechia; 3 First Department of Neurology, Faculty of Medicine, Masaryk University and St. Anne’s University Hospital, Brno, Czechia

**Keywords:** Professionalism, Self-compassion, Good medical practice, Medical students

## Abstract

**Background:**

To be a “good doctor” and have “good medical practices” are apparent goals for both medical students and medical faculties. However, the associated implicit and explicit standards could be a source of distress in the form of pressure to achieve professionalism. Self-compassion has been identified as a transtherapeutic factor that plays a crucial role in developing and maintaining mental health. It seems to be an essential meta-skill to learn, especially for medical students who often perceive imperfection as failure. In this pilot study, we investigated the qualities that medical students attribute to the “good doctor” concept, how they perceive themselves compared to this concept, and whether any possible discrepancy between these two perspectives could be associated with self-compassion.

**Methods:**

Altogether, 301 medical students participated in the study (mean age 22.3 ± 2.1; 71.8 % female). The discrepancy between concepts was measured by a semantic differential consisting of a list of 36 adjectives and antonyms that students repeatedly mentioned in courses in their responses to the question “What should a doctor be like?” Self-compassion was measured by the Self-Compassion Scale.

**Results:**

The obtained results offer an insight into students’ conceptualization of a “good doctor” and the hierarchy of given characteristics. Statistical analysis revealed significant associations between the discrepancy between the “ideal” doctor concept vs. actual self-perception and Self-Compassion Scale scores. The more students are compassionate to themselves, the lower the discrepancy.

**Conclusions:**

The current pilot study supports the hypothesis that student self-compassion could play some role in the degree of discrepancy between the ideal “good doctor” image and student self-concept. This result could support the importance of educational interventions developing self-compassion for medical students. The proposed discrepancy measurement could also be a tool for measuring the effect of well-being programs aimed at self-compassion in medical students.

**Supplementary Information:**

The online version contains supplementary material available at 10.1186/s12909-021-02930-2.

## Background

Getting accepted into medical school and becoming a doctor is a dream for many students. For those who have thought about the idea of practising medicine from as early as middle school or even grade school, doctoring fulfils a calling, and medicine is fascinating for them. The motivation behind the study of medicine and life´s work is complex. It includes various needs and motives such as interest in science, desire to help people, influence and social respect, job stability, high salary, and exciting job. In this context, most medical students want to be a “good doctor” and have “good medical practices” in their future to fulfil the notion they have dreamed about. This notion is based on the integration of their personal motives and needs and professional standards recommended by medical associations (e.g., [1]), public bodies protecting patient safety and improving medical education (e.g., [2]), hidden curricula [3], and competency-based medical education [4], and of patient needs and preferences [5]. Facing these intrinsic and extrinsic forces in combination with demanding study at the Faculty of Medicine, the desire to be a „good doctor“ is not just the motivation for personal and professional growth, but it could also be a critical source of distress for many students in the case they are not able to fulfil it. From the perspective of Faculty of Medicine teachers, we are challenged to help our students to deal with this pressure to achieve professionalism and to help them to live with the discrepancies between their dreams, ideals and natural limits. This does not mean that students should not strive to grow professionally; however, they need to be equipped with psychological tools for learning to live with innate human imperfection under this continual pressure. In this context, self-compassion seems to be a universal skill helping us relate to ourselves warmly and with kindness when we experience difficulties, fail, or notice something we do not like about ourselves. However, the relationship between professionalism and self-compassion has not been addressed yet in the literature.

The clear operationalization of professionalism across several domains, such as knowledge, skills, and attitudes (including personal qualities and attributes), represents a key medical education challenge [6] to create the medical education programs to help students develop desirable qualities of future doctors. “Professionalism is the basis of medicine’s contract with society” [7]. As part of this effort, the Royal College of Physicians and Surgeons of Canada conducted extensive research [8] that included the views of medical education representatives, health professionals, affiliated stakeholders, and the general public. They defined seven key roles of the ideal doctor: Medical Expert, Communicator, Collaborator, Manager, Health Advocate, Scholar, and Professional. They suggest that all these roles overlap in one general factor and create a Medical Expert. Van de Camp and colleagues [9] describe the three general themes of professionalism: (a) interpersonal professionalism (e.g., communication skills, leadership, trust, educating patients); (b) public professionalism (e.g., professional awareness, technical competence, being knowledgeable); and (c) intrapersonal professionalism (e.g., self-awareness, maturity, morality). In their mixed-methods exploration of the notion of the “good doctor” among junior and prospective medical students, Maudsley and her team [10] found that students valued compassion, patient-centered care, and communication skills over clinical competence and knowledge.

These domains should fulfill the primary outcomes of the explicit and hidden curriculum in medical training. They also create the more or less clear goal that students want to approach as future professionals. However, these standards could also be a source of distress, especially in medical schools where the standard of being “faultless and flawless leaves students with the feeling that they are constantly falling short” [11]. This corresponds with the results of a study of medical student perfectionism, socially prescribed perfectionism, and impostor phenomenon [12], showing that the “perception that others expect a great deal of you and will criticize any signs of failure” was an important predictor of medical student distress. In this context, Hill and Curran [13] showed in their meta-analysis that perfectionistic concerns displayed medium and medium-to-large positive relationships with overall burnout and symptoms of burnout.

Self-compassion is a key construct within the field of self-care that has been defined as relating to oneself with compassion by actively encouraging the expression of warmth, concern, and caring toward the self [14, 15]. The concept entails six components, put in three pairs: self-kindness vs. self-judgment, common humanity vs. isolation, and mindfulness vs. over-identification [15]. The first pair of opposites include a kind and understanding attitude to oneself when facing failure or one’s own inadequacy instead of harsh self-criticism. The second pair is about bearing in mind that making mistakes and being imperfect is an irreplaceable part of human life, and feelings of isolation are an inappropriate approach to one’s experience; in other words, it is necessary to accept that things do go wrong for most people at some point. The third pair focuses on a balanced view of one’s own thoughts and feelings. Self-compassion means to neither avoid nor over-identify with an immediate state of mind and emotions. Self-compassion, with all its benefits, can be trained. In a meta-analysis with an overall sample size of N = 16,416, a positive relationship was found between self-compassion and well-being, supporting the importance of self-compassion [16]. Weingartner and colleagues [17], in their Compassion Cultivation Training for medical students, showed that this kind of program is able to influence students’ self-compassion. Self-compassion has been associated with self-rated health in university students [18]. Self-compassion has been identified as a transtherapeutic and transdiagnostic phenomenon that plays a role in developing and maintaining mental health and quality of life [19, 20]. From this point of view, self-compassion is a critical meta-skill for medical students’ mental health and should be taught at medical schools.

In this context, we were interested in what qualities students attribute to the “good doctor” concept, how they perceive themselves compared to their “good doctor” concept, and whether any possible discrepancy between these two perspectives could be associated with self-compassion. We hypothesize that the discrepancy between what students think a good doctor should be like and how they perceive themselves is significantly negatively associated with their levels of self-compassion.

## Methods

### Sample and recruitment

Out of a total of 1910 medical students at the Faculty of Medicine (the academic year 2019/2020), 301 students participated in the survey (response rate 15.76 %; mean age 22.3 ± 2.1, 71.8 % female). The female to male ratio among Czech medical students is typically 2 to 1 [21].

The survey ran from January to the end of February 2020. All subjects were students of Masaryk University, recruited through advertisements on the website and the Facebook page of the Department of Psychology and Psychosomatics of the Faculty of Medicine. The inclusion criteria were that participants were medical students of the Faculty of Medicine of Masaryk University. The questionnaire directly asked which university they are studying at. Seventeen students were from other universities, and they were excluded from the total sample (N = 318).

No other exclusion criteria were applied. The survey was presented as a link to Google Forms. The survey was anonymous, and no personal data was collected. All students who participated in this study provided written informed consent for their anonymous participation. On the recommendation of the ethics committee of the Faculty of Medicine of Masaryk University and in accordance with the law, no ethical committee approval is necessary for this online, anonymous, and completely voluntary survey. All used methods were performed in accordance with the relevant guidelines and regulations.

### Polarities – Semantic differential

**Fig. 1:**
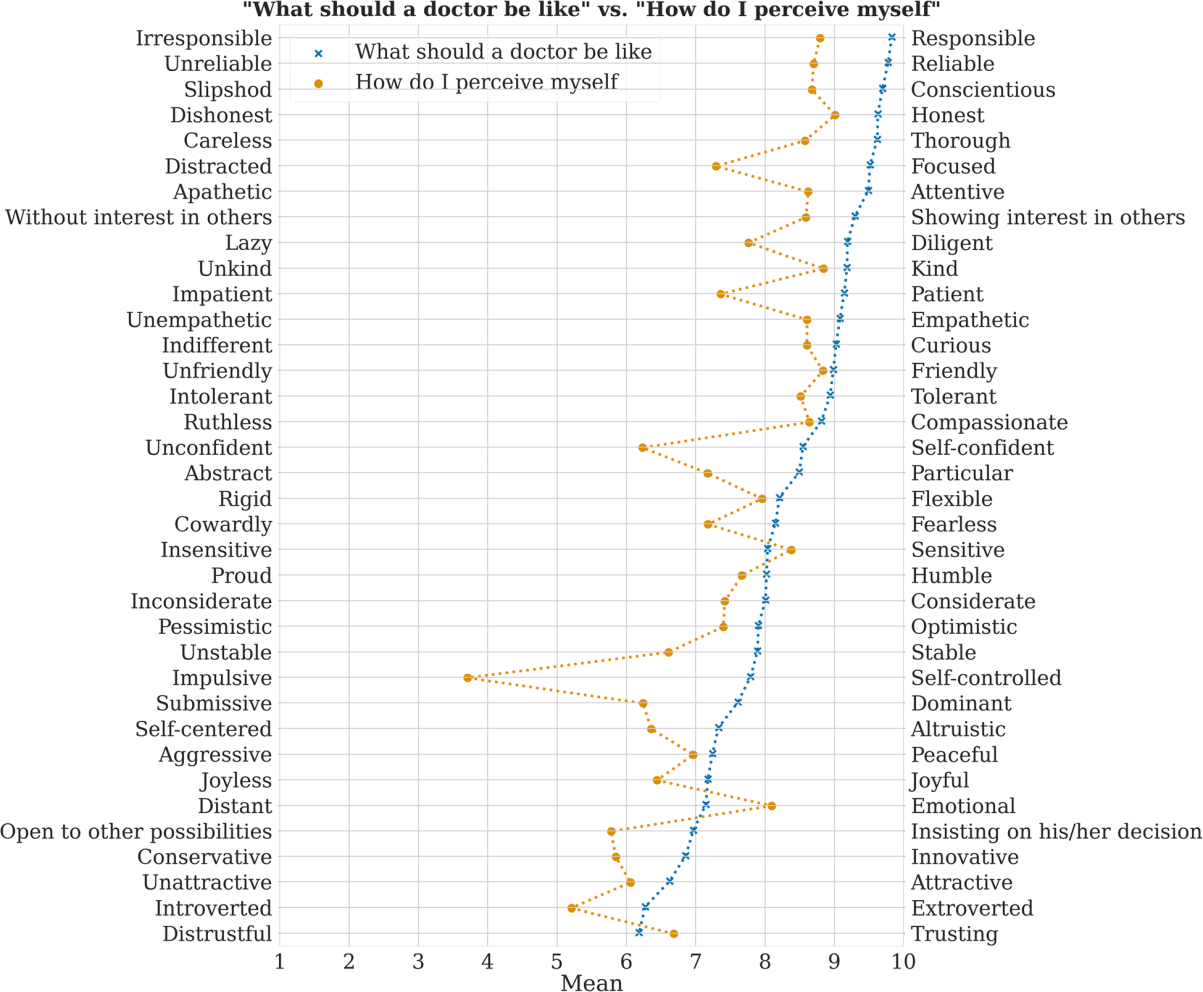
The differences between the “good doctor” concept vs. actual self-perception

We used a list of adjectives and their opposing words, that students repeatedly mentioned in their responses to the question “What should a good doctor be like?” during medical psychology seminars (Fig. 1, right column) within the years 2010–2016. After completing this list, students were asked to think of the opposite characteristics. Students’ answers were always written on the board by the teacher in the lecture. The list was discussed and students were given the opportunity to increase their “good doctor” concept awareness via the group discussion. This exercise was an integral part of the course “Medical Psychology and Psychosomatics”, which is compulsory for all students of general medicine in the fourth year of the study programme. This exercise aim is to help students to: (1) raise their awareness of which aspects they prefer in a “good doctor” concept; (2) discover the opposites of these characteristics (Fig. 1, left column); and (3) facilitate the process of integrating the conflicting forces between the often idealized notion of the “good doctor” and who they also are, such as ordinary imperfect human beings. The astonishment was their prevalent reaction across the study groups. They were surprised at how much is their “good doctor” notion idealized. The comment and explanation that they are still good and valuable persons even if they do not precisely fulfil all the requirements of the “good doctor” concept were often liberating for many of them.

A photo of the list on the board was always taken at the end of the lecture. The final polarities list used for this pilot study was composed of 50 sublists obtained from study groups in the mentioned time period (50 seminar groups of some 20 students). We got a list of 63 repeating words. The list has been reduced to 36 adjectives by the exclusion of synonyms by two independent teachers. The opposing words were modified in some cases using the Dictionary of Czech synonyms and antonyms. The original Czech version is attached for completeness in additional file 1. We used this list of polarities in our pilot research to quantify the degree of discrepancy between the “good doctor” concept and students’ actual self-perception on this continuum. This list has been developed specifically for this pilot study to test the study hypothesis. The list has not been published anywhere yet. It is still only an experimental version for the purpose of this pilot study. Each polarity was assessed on a nine-point scale (see Fig. 1). Whereas we have not used this exercise in this form for the last five years, only students who have not undergone this exercise earlier were included in this pilot study.

### Self-compassion

The Self-Compassion Scale [22], the Czech version [23], measures individual compassion for oneself. The SCS-SF is a short form of the 26-item Self-Compassion Scale (SCS) and has a high correlation with the full SCS (r ≥ 0.97; [14]. The SCS-SF has six subscales with two items in each subscale: Self-Kindness, Self-Judgment, Common Humanity, Isolation, Mindfulness, and Over-identified. The items are rated from 1 = “never” to 5 = “always.” The internal consistency of the subscales ranges between Cα = 0.65–0.86; total 0.89 [22]. A total self-compassion score is calculated by averaging the mean subscale scores after reverse coding the negative items. A study of diverse and international samples shows that the subscales are best explained by a general overall factor of self-compassion [24].

### Data analysis

Data analysis was conducted with Statistica 13.5 [25]. Figure 1 was created with Seaborn 0.10.1 [26] and Matplotlib 3.3.0 [27]. Even though the data are not precisely normally distributed (Shapiro-Wilk W = 0.92642, p < 0.001), the shape of the histogram for a given data set and the current sample size allow parametric statistics to be used. The relationship between the mean differences for each ideal vs. actual self-perceived characteristic and the self-compassion subscales was determined using the Pearson correlation. A simple linear regression was calculated to predict the role of self-compassion in the difference between ideal and self-perceived characteristics.

## Results

The descriptive statistics presented in Fig. 1 show the differences between the “good doctor” concept and actual self-perception (mean difference of 1.4 ± 0.47; min 0.3 max 3.5).

Correlation analyses revealed low to medium significant correlations in the difference between the “good doctor” concept vs. actual self-perception and the Self-Compassion Scale subscale scores (Table 1). The more the students relate to themselves with higher self-kindness (e.g., “I try to be loving toward myself when I’ m feeling emotional pain”), common humanity (e.g., “I try to see my failings as part of the human condition”), and mindfulness (e.g., “When something painful happens I try to take a balanced view of the situation”), the less the discrepancy between their “good doctor” concept and self-perception is.

Conversely, a more self-judgmental attitude to oneself (e.g., “I’ m disapproving and judgmental about my own flaws and inadequacies”), a tendency to react to suffering by isolation from others (e.g., “When I think about my inadequacies, it tends to make me feel more separate and cut off from the rest of the world”) and over-identification with the suffering (e.g., “When I’ m feeling down I tend to obsess and fixate on everything that’s wrong”) are more associated with the mentioned discrepancy.

**Table.1:** The correlations between the “good doctor” concept vs. self-perceived difference and self-compassion subscales

	Self-Kindness	Self-Judgment^b^	Common Humanity	Isolation^b^	Mindfulness	Over-identified^b^	Self-compassion total^a^
“good doctor” vs. self-perception difference	-0.32^***^	0.27^***^	-0.12^*^	0.36^***^	-0.13^*^	0.33^***^	-0.35^***^
Self-compassion total	0.77^***^	0.72^***^	0.58^***^	0.79^***^	0.68^***^	0.78^***^	-

*p < 0.05; **p < 0.01; ***p < 0.0001; ^a^the higher total score indicates higher self-compassion; ^b^self-judgment, isolation, and over-identification subscales were calculated by reverse coding: higher values mean lower self-judgment, isolation, and over-identification.

A significant regression equation was found (F (1,299) = 41.483, *p* < 0.000), with an R2 of 0.122. The students’ predicted ideal vs. self-perception difference is equal to 2.175–0.261 ( the total score of the Self-Compassion Scale). Thus, the difference between the ideal notion of how a good doctor should be and self-perception increases 0.261 for every point decrease in the Self-Compassion Scale score.

The statistical analysis did not reveal any significant difference between men (1.49 ± 0.50) and women (1.41 ± 0.46) in their “good doctor” concept vs. actual self-perception difference (t = 1.26; *p* > 0.21).

## Discussion

The current pilot study reveals that student self-compassion could play some role in the degree of discrepancy between their ideal image of a “good doctor” and their actual self-perception. While studies in this field often focus on a more general relationship between self-compassion, compassion fatigue, and burnout syndrome among healthcare professionals [28] or between self-compassion and well-being in medical students [29], our pilot study describes a new specific association between self-compassion and the “good doctor” concept. Following other studies [18, 30–33], our partial results could contribute to the discussion of the meaningfulness of educational interventions for medical students that develop self-compassion, such as mindfulness-based programs [34–36]. Although our current results look promising and open a relatively new question in this area, an essential part of the future work will entail incorporating other psychological concepts such as self-criticism, perfectionism, stress, shame and guilt and self-esteem.

The current data and their statistical analysis do not determine a causal relationship between students´self-compassion and discrepancy between their ideal image of a “good doctor” and their actual self-perception. The self-compassion could moderate the discrepancy and vice versa. We do not know from our current results how the students experience the referred discrepancy and if it is a source of positive motivation or personal distress. According to Higgins´s Self-Discrepancy theory [37], the discrepancy between our actual self-concept and, in our case, the ideal image of a “good doctor” can form a psychological background for the motivation to reach a condition where our self-concept matches our ideals, or it increases our vulnerability to dejection-related emotions such as disappointment, dissatisfaction, frustration, shame or embarrassment. Our experience is always the result of our interpretation of external and internal events. In this context, self-compassion, such as transtherapeutic and transdiagnostic phenomenon playing a role in developing and maintaining mental health and quality of life [19, 20], seems to be a critical meta-skill or interpretation filter helping students in some cases to relieve from dejection-related emotions associated with high discrepancy (self-concept versus the ideal image of a “good doctor”). This way of thinking could support the interpretation that self-compassion is mediating its association with the discrepancy. On the other hand, the perceived high discrepancy can activate a self-critical attitude that they do not deserve compassion. In this context, to test the hypothesis that the discrepancy is a source of distress and that self-compassion is a significant mediator of this relationship, future research will require to assess how do the students perceive the discrepancy in the interaction with the level of their perfectionism, self-criticism and self-reassurance.

As expected, the ideal characteristics were rated higher than the self-assigned characteristics. The exceptions to this expectation differed in three polarities: Insensitive – Sensitive, Distant – Emotional, and Distrustful – Trusting. These results correspond with our observations in discussions with students. They often state that to protect themselves from emotional exhaustion or burnout in clinical practice, a good doctor should be a little insensitive and distant. They also usually discuss their experience from hospital internships and observations that patients are not generally telling the truth (wittingly or unwittingly). To be a good doctor, they believe, you need to verify patient information, and then you need to be a little distrustful.

### Limits, future directions, and practical implications

Although our current results look promising and they open a relatively new question in this area, there are some limits of the study at this moment. Firstly, the used list of adjectives is not a standardized method for the exhaustive description of all characteristics which could be assigned to the “good doctor” concept. Next, using this list in different language environment should be done with caution because of possible translational shifts. The original Czech version of the list is attached as an additional file. The development of the standardized list of these adjectives validated, for instance, by “snowball technique”, structured by factor analysis and compared internationally, would be an appropriate next step. Finally, the current data and their statistical analysis do not determine a causal relationship between observed variables. Future research should investigate more complex psychological models, as was suggested above in the discussion.

## Conclusions

This study presents a new specific association between self-compassion and the “good doctor” concept. Its results indirectly support the assumption that self-compassion could relieve the distress associated with professionalism pressure. The proposed discrepancy measurement could be a tool for measuring the effect of well-being programs aimed at self-compassion in medical students.

## Supplementary Information

**Additional file 1.**

## Data Availability

The dataset generated and analyzed during the current study is available to the authors but is not publicly available due to ethical guidelines. The datasets used and/or analyzed during the current study available from the corresponding author on reasonable request.
