# Supplementary figures and images for: Self-compassion in medical students: a pilot study of its association with professionalism pressure
Source: BMC Med Educ. 2021 Sep 22;21:500. doi: 10.1186/s12909-021-02930-2 (PMC8455803; doi:10.1186/s12909-021-02930-2)

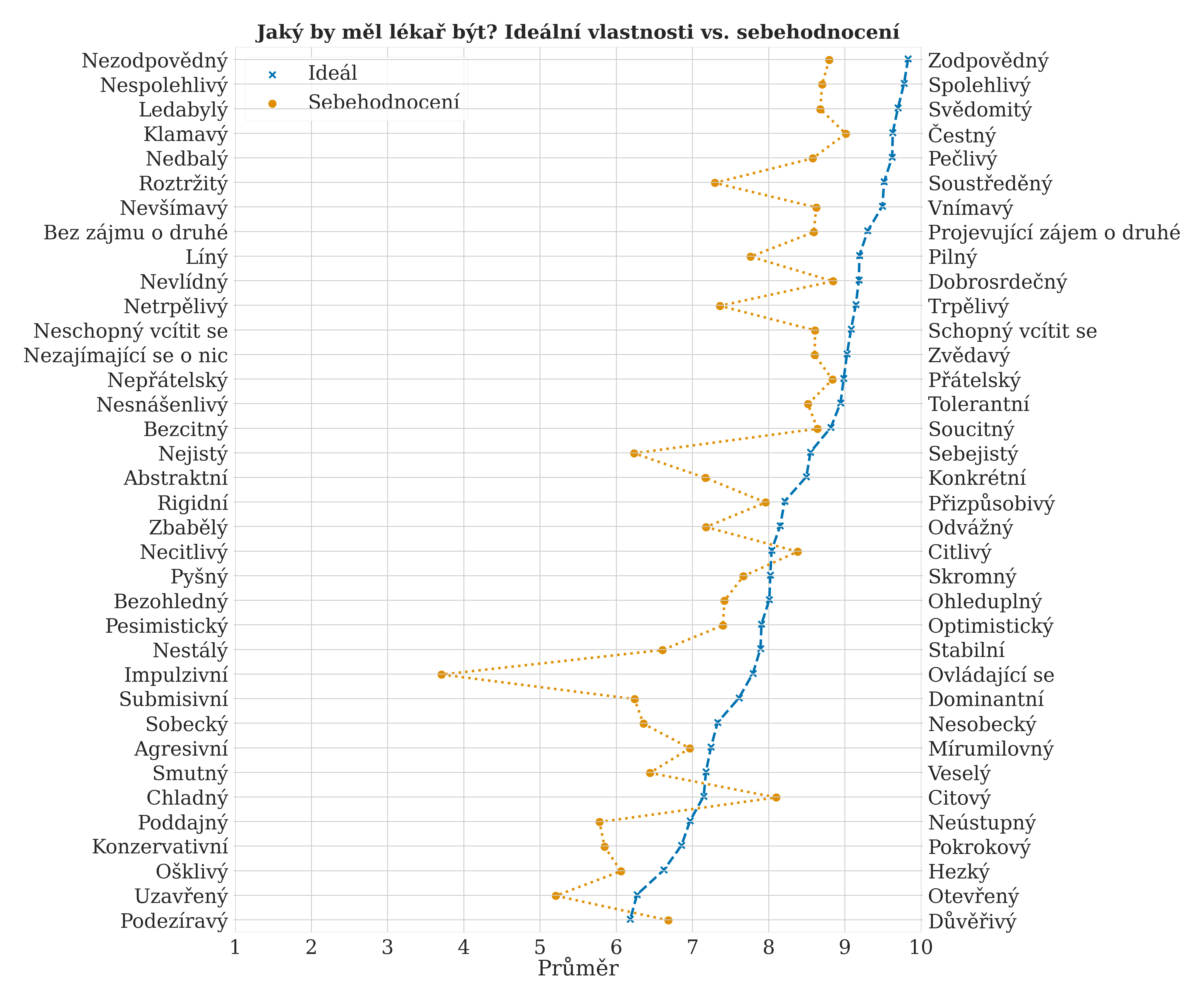

Supplement: Supplementary file 1 — Additional file 1. [file 12909_2021_2930_MOESM1_ESM.png]
